# Estimating standardized ileal digestible methionine requirements for gilts during gestation using whole-body nitrogen retention and describing plasma creatine, glutathione, and taurine concentrations

**DOI:** 10.1093/jas/skaf156

**Published:** 2025-05-04

**Authors:** Cristhiam Jhoseph Munoz Alfonso, Cierra Kozole, John Kyaw Htoo, Lee-Anne Huber

**Affiliations:** University of Guelph, Guelph, ON, Canada N1G 2W1; University of Guelph, Guelph, ON, Canada N1G 2W1; Evonik Operations GmbH, Hanau-Wolfgang, Germany; University of Guelph, Guelph, ON, Canada N1G 2W1

**Keywords:** gestating gilts, methionine requirement, whole-body nitrogen retention, plasma creatine

## Abstract

A total of 70 gestating gilts (166 ± 13 kg initial BW on day 31 of gestation) were used to determine the standardized ileal digestible (**SID**) Met needed to maximize whole-body N retention and to describe the impact of dietary Met on indicators of Met utilization for roles beyond protein synthesis. Seven days prior to N balance measurements between days 38 and 41, days 53 and 56, days 87 and 90, and days 109 and 112 of gestation (periods 1, 2, 3, and 4, respectively), gilts were assigned to 1 of 7 dietary treatments (*n* = 10) that titrated SID Met between 50% and 150% of perceived requirements of 3.5 and 5.2 g SID Met/d (before and after day 90 of gestation, respectively) using dl-Met and in the presence of excess Cys in an incomplete randomized block design. Fasted blood samples were collected on days 38, 53, 87, and 109 to assess concentrations of plasma Met-derived metabolites. Contrast statements were used to determine linear and quadratic effects of the dietary inclusion level of SID Met and quadratic polynomial (**QPM**), broken-line linear, and broken-line quadratic (**BLQ**) ascending models were evaluated for the primary response variables. Whole-body N retention did not exhibit linear or quadratic relationships with increasing SID Met content in periods 1, 2 and 3, but inflection points were observed at 0.17% (3.8 g SID Met/d; QPM), 0.19% (4.2 g SID Met/d; BLQ), and 0.16% SID Met (3.5 g SID Met/d; QPM), respectively. Whole-body N retention increased (linear and quadratic; *P* < 0.001) with increasing SID Met content in period 4, optimized at 0.23% (6.0 g SID Met/d; QPM). Plasma creatine concentration did not exhibit linear or quadric relationships with increasing SID Met contents, but the QPM indicated inflection points at 0.17%, 0.17%, 0.22%, and 0.18% SID Met (3.8, 3.8, 4.9, and 4.7 g SID Met/d) on gestation days 38, 53, 87, and 109, respectively. Plasma concentrations of GSH tended to increase then decrease (quadratic; *P *= 0.096) on day 53 and tended to increase (linear; *P *= 0.095) on day 87 as SID Met increased; inflection points were observed at 0.16%, 0.17%, 0.21%, and 0.16% SID Met (3.5, 3.8, 4.6, and 4.2 g/d; QPM) on gestation days 38, 53, 87, and 109, respectively. Thus, the SID Met feeding recommendations provided by the NRC (NRC 2012. Nutrient requirements of swine. 11th rev ed. Washington D.C.: National Academy Press) gestating sow model are insufficient to maximize whole-body N retention of gilts throughout gestation in current production conditions, while the supply of SID Met might also influence Met utilization for metabolic fates beyond protein retention.

## Teaser

The feeding recommendations for Met provided by the NRC (NRC 2012. Nutrient requirements of swine. 11th rev ed. Washington D.C.: National Academy Press) gestating sow model are insufficient to maximize whole-body N retention of gilts at all stages of gestation in current production conditions. The supply of dietary Met may also influence Met utilization for metabolic fates beyond protein retention in the gestating gilt.

## Introduction

The amino acid (**AA**) requirements of sows are dynamic throughout gestation and are currently based on estimated protein retention rates and AA profile of 5 protein-containing pools (fetuses, mammary tissue, placenta and associated fluid, uterus, and maternal body) in the NRC (2012) gestating sow model. In developing the NRC (2012) gestating sow model, empirical estimations of AA requirements published in the scientific literature were used to evaluate the model but for the sulfur amino acids (**SAA**), Met and Cys, only 2 requirement studies for gestating sows met the inclusion criteria (i.e., Rippel et al., 1965; Holden et al., 1971). These studies, however, were conducted with sows that had total litter sizes of 8.56 and 7.60 piglets, respectively, which are not representative of the current performance expectations for modern sow genetic lines. Therefore, to ensure that the nutrient requirement model is still applicable for the performance levels and physiology of modern gestating sows, despite the ability to scale estimated litter size in the current NRC (2012) model, updated empirical studies are required.

Many amino acids fulfill roles in the body beyond serving as precursors for protein synthesis (Batista‐Silva et al., 2019; Sivanand and Vander Heiden, 2020; Alagawany et al., 2021). In particular, Met is the main precursor of the methyl donor, S-adenosyl-methionine (**SAM**), which participates in a number of transmethylation reactions (Lu, 2000). Among these reactions, creatine (intracellular energy source and neurotransmitter; Brosnan and Brosnan, 2016) and phosphatidylcholine (**PC**; involved in lipid metabolism and endogenous recycling of the methyl donor betaine; Lordan et al., 2017) synthesis use a majority of methyl groups during neonatal growth (Robinson et al., 2016a). The methylation of DNA to control gene expression is a mechanism of fetal programming and epigenetic transformation, although this transmethylation process uses less than 1% of Met supply in neonatal piglets (Bertolo and McBreairty, 2013). In instances of perturbed dietary Met supply, the partitioning of Met between protein synthesis and transmethylation reactions can be altered, and in neonatal piglets, the amount of Met required to maximize the synthesis of creatine and PC was greater than that for protein synthesis (Robinson et al., 2016b). Thus, the use of Met for non-proteinogenic fates may be important for the transmethylation pathway, since SAM is synthesized endogenously. Moreover, homocysteine (**Hcys**) is a co-product of transmethylation and can be re-methylated to re-form Met (Raghubeer and Matsha, 2021) or move through the transsulfuration pathway (irreversible) to synthesize Cys (Finkelstein and Martin, 2000). Cysteine is necessary for protein synthesis, but also for the production of glutathione (**GSH**) and taurine (**Tau**; McBean, 2012), which are important for detoxification of oxidative agents (Adeoye et al., 2017), among other functions. The inclusion of exogenous Cys can spare up to 40% of the need for dietary Met (Shoveller et al., 2022). Therefore, the dietary supply of Met is important for protein synthesis, but also to participate in transmethylation and transsulfuration (via Hcys) pathways to generate additional necessary molecules.

Therefore, the hypothesis was that dietary Met required to optimize whole-body N retention during gestation will be relatively greater than current feeding recommendations due to greater productivity of modern genetics. Moreover, indicators of Met utilization for nonprotein fates will be influenced by dietary Met supply since Met utilization for transmethylation was not considered in the current Met requirement estimates for gestating gilts. The objective of the current study was to evaluate the current NRC (2012) recommendation for Met for gilts throughout gestation using whole-body N retention and to describe the impact of dietary Met inclusion level on indicators of Met utilization for roles beyond protein synthesis.

## Materials and Methods

Animal care and use protocols were approved by the University of Guelph Animal Care and Use Committee (AUP #4172). Animals were cared for in accordance with the Canadian Council on Animal Care guidelines (CCAC, 2009).

### Animal housing, experimental design, and diets

A total of 70 gilts (Yorkshire × Landrace; 166 ± 13 kg initial BW at 31 d of gestation) were used in the experiment at the Arkell Swine Research Station (Guelph, ON, Canada) over 5 blocks (breeding batches). Gilts were selected once pregnancy was confirmed via ultrasound approximately 28 d after breeding, housed in groups, and received a commercial gestation diet. Seven days prior to N balance measurements between days 38 and 41, days 53 and 56, days 87 and 90, and days 109 and 112 of gestation (periods 1, 2, 3, and 4, respectively), gilts were moved to individual stalls and assigned to 1 of 7 dietary treatments (*n* = 10; Table 1) in an incomplete randomized block design. The isocaloric experimental diets were formulated using faba beans, wheat, field peas, soybean hulls, and cornstarch and provided standardized ileal digestible (**SID**) Met at 50%, 66.7%, 83.3%, 100%, 116.7%, 133.3%, and 150% of the currently perceived Met requirement for gestating gilts with 145 kg of body weight at breeding, an anticipated litter size of 12.3 piglets, and 1.35 kg piglet birth weight (NRC, 2012) by replacing cornstarch with dl-Met (98% purity). The currently perceived Met requirements were 3.5 and 5.2 g SID Met/d before and after day 90 of gestation, respectively (NRC, 2012). Thus, first set of 7 diets was formulated for periods 1, 2, and 3 (days 38 to 41, days 53 to 56, and days 87 to 90 of gestation) and a second set of 7 diets was formulated for period 4 (days 109 to 112 of gestation) due to greater estimated nutrient requirements in late gestation (NRC, 2012). Feed allowance was also different for periods 1, 2, and 3 (2.21 kg/d) vs. period 4 (2.61 kg/d) but similar to the standard gestation diet allowance. All indispensable AA were provided at least 20% above estimated SID requirements and choline, folate, and other vitamins such as B12 were included to meet the estimated requirements for gestating sows (NRC, 2012). Feather meal was included in the dietary treatments as a protein source with relatively low Met but high enrichment of Cys; l-Cys was also included to minimize Met utilization for transsulfuration. Titanium dioxide was included at 0.10% as an indigestible marker in all experimental diets. Experimental diets were provided in 2 equal meals per d at 700 h and 1,500 h during adaptation (7 d) and the N balance collection period (4 d). Beyond the adaptation and N balance periods, gilts returned to group housing and again received the commercial gestation diet.

**Table 1.** Ingredient composition and calculated nutrient contents of experimental diets (as-fed basis)^1^^,^^2^

	Basal diet 1	Basal diet 2
Ingredient composition, %		
Wheat, soft red	5.00	5.00
Field peas	10.00	10.00
Faba beans	20.00	20.00
Soybean hulls	20.00	20.00
Cornstarch	36.01	29.15
Feather meal^3^	4.60	8.20
Animal and vegetable fat blend	—	2.50
Vitamin and mineral premix^4^	1.50	1.50
Monocalcium phosphate	0.93	1.15
Limestone	0.70	0.85
Sugar	0.40	0.40
Choline chloride 60%^5^	0.32	0.32
l-Lys-HCl	0.19	0.42
dl-Met	—	—
l-Thr	0.12	0.16
l-Trp	0.04	0.08
l-Cys	0.06	0.10
l-His	0.03	0.07
Titanium dioxide	0.10	0.10
Total	100.00	100.00
Calculated nutrient contents		
Crude protein, %	14.14	17.34
Net energy, Kcal/kg	2,486	2,481
Calcium, %	0.94	1.04
Total phosphorus, %	0.50	0.56
Standardized total tract digestible P, %	0.41	0.46
Total Lys, %^6^	0.89 (0.61)	1.19 (0.82)
Total Met, %	0.11 (0.08)	0.14 (0.10)

^1^Basal diet 1 was used in experimental periods 1, 2, and 3 and fed between gestation days 31 and 41, days 46 and 56, and days 80 and 90. Basal diet 2 was used in experimental period 4 and fed between gestation days 102 and 112.

^2^Experimental diets were formulated to be identical within period, with the exception of dl-Met that was added at the expense of cornstarch in increments in the basal diets to provide 7 levels of SID Met between 50% and 150% of estimated Met requirements. For experimental diets fed in periods 1, 2, and 3, dl-Met was added between 0% and 0.16% inclusion, for experimental diets fed in period 4, dl-Met was added between 0% and 0.23% inclusion.

^3^Feather meal (Sanimax Inc, Guelph, ON, Canada).

^4^Provided per kg of premix: vitamin A, 254,147 IU; vitamin D3, 45,133 IU; vitamin E, 2,467 IU; vitamin K, 63.5 mg as menadione; pantothenic acid, 800 mg; riboflavin, 211.8 mg; folic acid, 74.1 mg; niacin, 1,059 mg; thiamin, 63.5 mg; pyridoxine, 84.7 mg; vitamin B_12_, 847.3 µg; biotin, 11.3 mg, as biotin 2%; Cu, 529.5 mg; Fe, 4,533 mg; Mn, 1,271 mg; Zn, 5,667 mg, as Zinc oxide 72%; Se, 8.7 mg; I, 21.2 mg; Salt 267 g; and Limestone, 673 g. (Wallenstein Feed & Supply Ltd., Wallenstein, ON, Canada).

^5^Choline chloride 60% (Pestel Nutrition Inc., New Hamburg, ON, Canada).

^6^SID values are provided in parentheses.

### Sample collection

During each N balance period, total daily urine output was collected using urinary catheters (Lubricath®, 2-way, 30 cc balloon, 18 FR, Bard Medical Canada Inc., Oakville, ON, Canada) and feces were grab-sampled, as described previously (Huber et al., 2015). Urine was collected into a container with 50 mL of 20% v/v sulfuric acid to reduce N volatilization. After each successful 24-h collection, a representative subsample of 5% (wt) was obtained, pooled per gilt and per N balance period, and stored at 4^o^C. At the end of the N balance period, the urinary catheter was removed, the pooled urine aliquots were mixed thoroughly, and 2 subsamples were collected and frozen at −20 °C until further analysis (Miller et al., 2016). Fresh and uncontaminated feces were manually collected daily, pooled per gilt and per N balance period, and frozen at −20 °C until further analysis. At the beginning of each N balance period and 15 h after the last meal, gilts were weighed and 16 mL of blood was collected via the orbital sinus (Dove and Alworth, 2015). Six milliliters were collected into vacutainer tubes coated with lithium heparin (Becton Dickinson & Co, Franklin Lakes, NJ) for plasma collection, and 10 mL of blood were collected into untreated tubes (BD vacutainer; Franklin Lakes, NJ, USA) for serum collection. All blood samples were separated by centrifugation at 3,430 × g for 15 min at 4 ^o^C and stored at −80 °C until further analyses.

### Chemical analyses

The experimental diets were analyzed in quadruplicate for dry matter (Method 930.15; AOAC, 2019) and ash (method 942.05; AOAC, 2019), and in duplicate for crude protein (N × 6.25; Method 990.03; AOAC, 2019) using the combustion method (LECO FP628; LECO CORPORATION, Saint Joseph, MI). The AA composition of the experimental diets was determined by Evonik Operations Gmbh (Hanau, Frankfurt, Germany) using exchange chromatography with post-column derivatization with ninhydrin after hydrolysis with 6 N HCL for 24 h at 110 °C. The AA were oxidized with performic acid, which was neutralized with sodium metabisulfite (Llames and Fontaine, 1994; Commission Directive, 1998). Tryptophan was determined by HPLC with fluorescence detection (extinction 280 nm, emission 356 nm), after alkaline hydrolysis with barium hydroxide octahydrate for 20 h at 110 °C (Commission Directive, 2000). Tyrosine was not determined. Gross energy was analyzed in duplicate using a bomb calorimeter (IKA Calorimeter System C 5000; IKA Works Inc., Wilmington, NC) and benzoic acid was used as the standard for calibration (Tables 2 and 3). Fecal samples were freeze dried and finely ground prior to analyses of dry matter, ash, and N (as above) and titanium content in experimental diets (quadruplicate) and fecal samples (triplicate) were analyzed following procedure described by Christensen and Huber (2021). The N content of urine was also analyzed (singlicate), as described above.

**Table 2.** Analyzed nutrient contents in the experimental diets fed for periods 1, 2, and 3 (as-fed basis)

	SID Met, %^1^						
Item	0.08	0.11	0.13	0.16	0.19	0.21	0.24
Crude protein, %	13.05	13.69	13.37	13.27	14.19	13.52	13.49
Gross energy, Kcal/kg	3,757	3,788	3,700	3,725	3,802	3,765	3,820
Indispensable amino acids, %							
Arg	0.87	0.89	0.91	0.92	0.97	0.95	0.94
His	0.28	0.28	0.29	0.29	0.31	0.30	0.29
Ile	0.52	0.53	0.53	0.54	0.57	0.55	0.56
Leu	0.93	0.94	0.94	0.96	1.00	0.97	0.98
Lys	0.77	0.80	0.82	0.83	0.88	0.86	0.89
Met	0.11	0.13	0.15	0.19	0.21	0.24	0.26
Phe	0.55	0.56	0.55	0.57	0.60	0.58	0.58
Thr	0.60	0.62	0.59	0.60	0.67	0.63	0.66
Trp	0.16	0.16	0.16	0.16	0.16	0.15	0.15
Val	0.65	0.66	0.66	0.69	0.72	0.69	0.72
Dispensable amino acids, %							
Ala	0.55	0.55	0.55	0.57	0.60	0.57	0.57
Asp	1.12	1.15	1.17	1.19	1.25	1.22	1.14
Cys	0.36	0.35	0.36	0.34	0.37	0.35	0.44
Glu	1.78	1.78	1.80	1.87	1.95	1.91	1.82
Gly	0.75	0.77	0.75	0.78	0.83	0.77	0.83
Pro	0.77	0.78	0.78	0.76	0.78	0.76	0.84
Ser	0.84	0.85	0.84	0.84	0.90	0.86	0.95

^1^One of seven diets were fed between gestation days 31 and 41 (period 1), days 46 and 56 (period 2), and days 80 and 90 (period 3) with SID Met inclusion of 50%, 66.7%, 83.3%, 100%, 116.7%, 133.3%, and 150% of NRC (2012) recommendations between days 30 and 89 of gestation.

**Table 3.** Analyzed nutrient contents in the experimental diets fed for period 4 (as-fed basis)

	SID Met, %^1^						
Item	0.10	0.13	0.17	0.20	0.23	0.27	0.30
Crude protein, %	17.13	16.83	17.71	16.89	17.34	17.36	17.85
Gross energy, Kcal/kg	3,886	3,811	3,920	3,850	3,877	3,908	3,940
Indispensable amino acids, %							
Arg	1.17	1.27	1.28	1.24	1.18	1.20	1.37
His	0.35	0.38	0.35	0.36	0.38	0.36	0.37
Ile	0.66	0.70	0.67	0.70	0.68	0.66	0.71
Leu	1.17	1.27	1.18	1.23	1.20	1.18	1.27
Lys	1.06	1.17	1.12	1.11	1.08	1.10	1.17
Met	0.17	0.22	0.23	0.25	0.28	0.31	0.35
Phe	0.68	0.74	0.69	0.72	0.72	0.69	0.75
Thr	0.77	0.85	0.79	0.79	0.78	0.81	0.83
Trp	0.21	0.22	0.23	0.22	0.21	0.22	0.22
Val	0.86	0.90	0.88	0.91	0.84	0.86	0.94
Dispensable amino acids, %							
Ala	0.69	0.75	0.69	0.72	0.70	0.69	0.74
Asp	1.37	1.48	1.40	1.44	1.51	1.38	1.48
Cys	0.51	0.57	0.52	0.53	0.43	0.52	0.53
Glu	2.20	2.38	2.23	2.28	2.36	2.22	2.35
Gly	0.98	1.06	0.98	1.02	0.98	0.98	1.06
Pro	0.99	1.08	0.98	1.04	0.95	1.01	1.07
Ser	1.11	1.25	1.13	1.17	1.07	1.15	1.23

^1^One of seven diets were fed between gestation days 102 to 112 (period 4) with SID Met inclusion of 50%, 66.7%, 83.3%, 100%, 116.7%, 133.3%, and 150% of NRC (2012) recommendations estimated between day 90 and 114 of gestation.

Blood plasma samples were analyzed for concentrations of dispensable and indispensable AA, Hcys, Tau, and GSH using ultra-performance liquid chromatography (**UPLC**; Banton et al., 2021). All AA were analyzed using 10% sulfosalicylic acid and norvaline as internal standard (Method 994.12; AOAC, 2019; Waters Corporation, Milford, MA). Total plasma Hcys, cysteinyl-glycine, and GSH were measured according to previously published methods (Vester and Rasmussen, 1991; Pfeiffer et al., 1999), with minor adaptations as described by Banton et al. (2021). The derivatized thiols were separated using an Acquity UPLC BEH C18 column (2.1 × 50 mm, 1.7 μm; Waters Corporation, Milford, MA) maintained at 28 °C with fluorescence detection at 515 nm emission and 385 nm excitation. The peak areas obtained were compared with known standards and analyzed using the Waters Empower 2 Software (Waters Corporation, Milford, MA).

Plasma creatine concentrations were analyzed using high performance liquid chromatography (Aligent Technologies, Santa Clara, CA) originally described by Lamarre et al. (2010), but with minor adaptations as described by Banton et al. (2022). The separation of creatine was performed in a Hypercarb column (4.6 × 100 mm, 5 um; Fisher Scientific, Ottawa, ON) using UV detection (210 nm) and a mobile phase of 3% acetonitrile and 0.1% trifluoracetic acid at a flow rate of 0.8 mL/min. The creatine peaks were compared to known standards (Sigma-Aldrich, St. Louis, MO).

Serum urea N (**SUN**) was determined using calorimetric methodology (Gregory et al., 2023) and following manufacturer recommendations (Sekisui diagnostic, Tokyo, Japan). The intra- and inter-assay CVs were 1.20% and 9.60%, respectively.

### Calculations and statistical analysis

Whole-body N retention was calculated according to Miller et al. (2016). Nitrogen intake was calculated in all periods by multiplying the analyzed N content of the respective diet by the feed allowance. Feed refusal was not predominant over the course of the experiment; for the few cases where feed was not consumed completely, the data were excluded from N balance calculations. Since there was only one batch of diets mixed, each treatment had a single value for the N intake and thus, standard error was not calculated. Normality and homogeneity of variances among treatments were assessed using the PROC UNIVARIATE statement of SAS® software (SAS Inst. Inc., Cary, NC). Outcomes were analyzed using the GLIMMX procedure of SAS with dietary Met level as the main effect and block and gilt within treatment as the random effects. Contrast statements were used to determine linear and quadratic effects of the inclusion level of Met on the response variables. Since the shapes of the response curves could differ from the standard linear and quadratic relationships, quadratic polynomial (**QPM**), broken-line linear, and broken-line quadratic (**BLQ**) ascending models (Gonçalves et al., 2016) were assessed using the Bayesian information criteria for the key response variables to meet the objectives of the study (namely whole-body N retention and plasma creatine and GSH concentrations), regardless if the linear and quadratic contrasts were significant. Statistical significance and tendencies were considered at *P* < 0.05 and 0.05 ≤ *P* < 0.10, respectively.

## Results

Analyzed crude protein contents aligned with calculated values for all experimental diets (Tables 1 to 3). For the experimental diets fed in periods 1 to 3, analyzed Met aligned with calculated values (Tables 1 and 2) but for diets fed in period 4, Met contents were greater than anticipated (Tables 1 and 3) for the diet with the lowest Met inclusion (0.14% calculated vs. 0.17% analyzed), which may have been due to an underestimation of Met supplied from feather meal during diet formulation. Nevertheless, when contemplating all analyzed vs. calculated values for total Met, the separation among treatments allowed for the study objectives to be achieved. There were also slight differences between calculated values and analyzed values for dispensable and indispensable AA (i.e., Lys), however, the indispensable AA were still supplied at or above estimated requirements.

### Nitrogen balance

**Figure 1.**
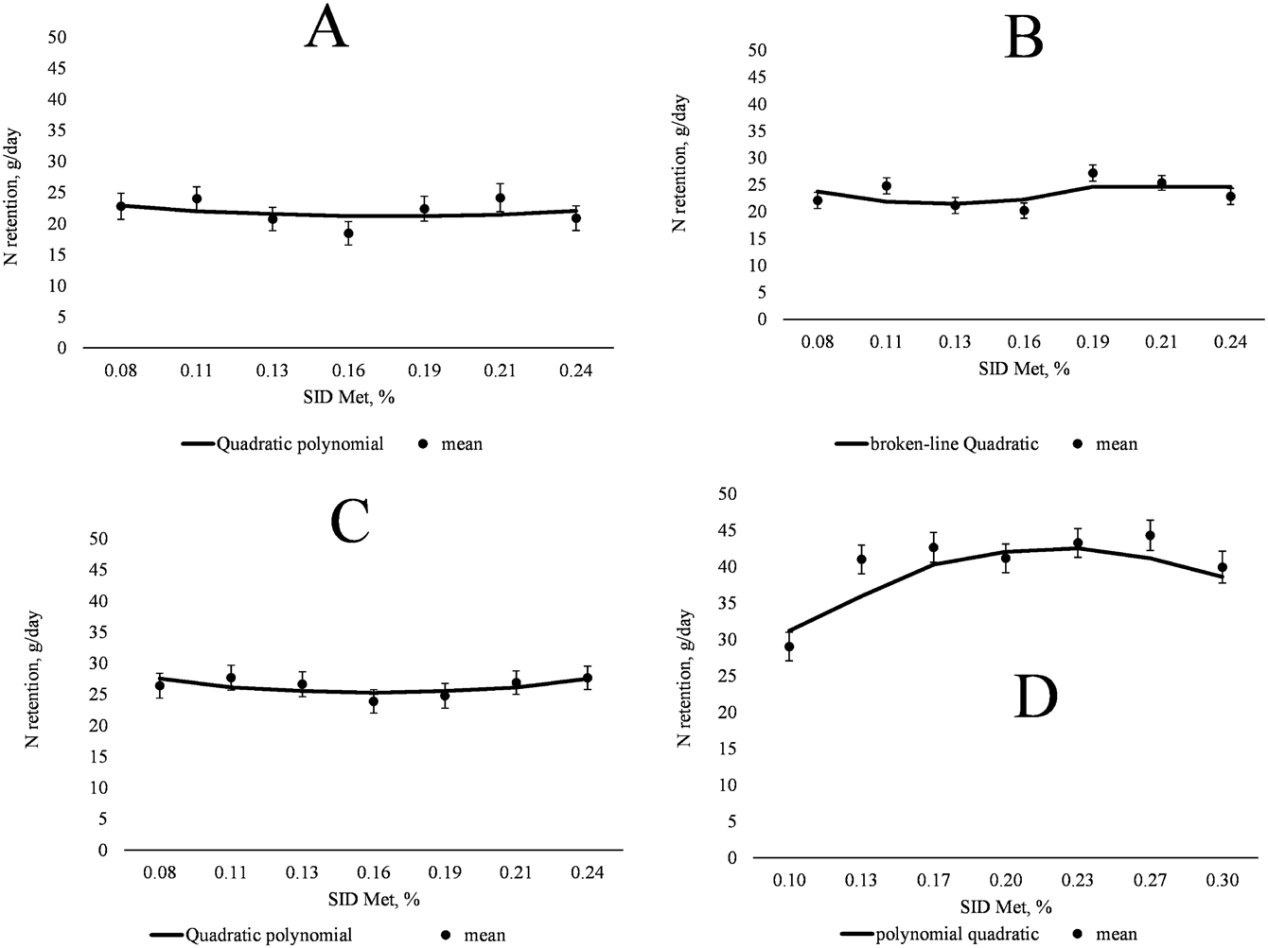
(A) Quadratic polynomial model for the relationship between nitrogen retention (g/d) and SID Met (% of diet) of gilts from days 38 to 41 of gestation (*n* = 10 for each treatment). N retention, g/d = 27.2 + (−68.6) × (SID Met, %) + 197.7 × (SID Met, %)^2^; Bayesian information criterion (BIC) = 412.3; inflection point at 0.17% SID Met. (B) BLQ model for the relationship between nitrogen retention (g/d) and SID Met (% of diet) of gilts from days 53 to 56 of gestation (left to right; *n* = 10, 9, 9, 10, 10, 9, and 10 respectively). N retention, g/d = 24.6 − (−106.3) × (0.19 − SID Met, %) + 892.6 × (0.19 − SID Met, %)^2^ if SID Met, % < 0.19; 24.6 if SID Met, % > 0.19. BIC = 353.8; Nitrogen retention optimized at 0.19% SID Met. (C) Quadratic polynomial model for the relationship between nitrogen retention (g/d) and SID Met (% of diet) of gilts from days 87 to 90 of gestation (left to right; *n* = 10, 9, 9, 9, 10, 10, and 10 respectively). N retention, g/d = 34.4 + (−115.1) × (SID Met, %) + 359.11 × (SID Met, %)^2^; BIC = 377.1; inflection point 0.16% SID Met. (D) Quadratic polynomial model for the relationship between nitrogen retention (g/d) and SID Met (% of diet) of gilts from days 109 to 112 of gestation (left to right; *n* = 8, 9, 10, 9, 10, 10, and 9 respectively). N retention, g/d = 4.4 + 349.6 × (SID Met, %) + (−767.7) × (SID Met, %)^2^; BIC = 377.3; Nitrogen retention optimized at 0.23% SID Met.

Between days 38 and 41 of gestation, fecal N excretion tended to increase (linear and quadratic; *P *= 0.082 and *P *= 0.076, respectively) and total N excretion tended to increase then decrease (quadratic; *P *= 0.051) with increasing dietary SID Met content (Table 4). Whole-body N retention did not exhibit a linear or quadratic relationship with increasing SID Met content, but the QPM indicated an inflection point at 0.17% SID Met (3.8 g SID Met/d; Fig. 1, panel A). Between days 53 and 56 of gestation, fecal N excretion increased then decreased (quadratic; *P *< 0.001) with increasing dietary SID Met content, while urinary and total N excretion were not influenced by dietary SID Met content. Whole-body N retention did not exhibit a linear or quadratic relationship with increasing SID Met content, but the BLQ model indicated an optimum at 0.19% SID Met (4.2 g SID Met/d; Fig. 1, panel B). Between days 87 and 90, fecal N excretion increased then decreased (linear and quadratic; *P *< 0.05) and total N excretion tended to increase then decrease (quadratic; *P *= 0.082) with increasing dietary SID Met content. Whole-body N retention did not exhibit a linear or quadratic relationship with increasing dietary SID Met content, but the QPM indicated an inflection point at 0.16% SID Met (3.5 g SID Met/d; Fig. 1, panel C). Between days 109 and 112, total (linear and quadratic; *P *< 0.05) and fecal (linear and quadratic; *P *< 0.05) N excretion decreased while urinary N excretion decreased then increased (quadratic; *P *< 0.001) such that whole-body N retention increased (linear and quadratic; *P *< 0.05) with increasing dietary SID Met content (Table 5). The QPM indicated that whole-body N retention was optimized at 0.23% SID Met (6.0 g SID Met/d; Fig. 1, panel D).

**Table 4.** Nitrogen utilization in gestating gilts fed diets containing 0.08%, 0.11%, 0.13%, 0.16%, 0.19%, 0.21%, or 0.24% SID Met between days 38 and 41, days 53 and 56, or days 87 and 90 of gestation

	SID Met, %							SEM^1^	*P*-value^2^	
	0.08	0.11	0.13	0.16	0.19	0.21	0.24		Linear	Quadratic
Period 1 (days 38 to 41)										
Number of sows^3^	10	10	10	10	10	10	10			
N intake, g/d	46.14	48.41	47.29	46.96	50.18	47.83	47.72			
Total N excretion, g/d	20.82	24.19	26.55	28.48	27.80	23.59	26.91	2.10	0.113	0.051
Urinary N excretion, g/d	13.85	12.42	14.83	14.06	13.95	12.05	13.77	2.34	0.865	0.808
Fecal N excretion, g/d	11.47	11.63	11.72	14.42	14.14	11.91	12.99	0.77	0.082	0.076
Whole-body N retention, g/d	22.79	24.04	20.74	18.45	22.4	24.17	20.88	2.07	0.730	0.417
Period 2 (days 53 to 56)										
Number of sows^3^	10	9	9	10	10	9	10			
N intake, g/d	46.14	48.41	47.29	46.96	50.18	47.83	47.72			
Total N excretion, g/d	24.05	23.60	26.13	26.74	23.00	22.46	24.88	1.68	0.683	0.438
Urinary N excretion, g/d	13.48	10.52	14.22	13.36	10.04	11.19	14.19	2.04	0.935	0.428
Fecal N excretion, g/d	9.86	12.74	12.12	13.20	13.17	11.27	10.90	0.65	0.667	< 0.001
Whole-body N retention, g/d	22.09	24.80	21.16	20.19	27.18	25.37	22.84	1.68	0.195	0.930
Period 3 (days 87 to 90)										
Number of sows^3^	10	9	9	9	10	10	10			
N intake, g/d	46.14	48.41	47.29	46.96	50.18	47.83	47.72			
Total N excretion, g/d	19.75	20.72	20.64	23.07	25.40	20.93	20.05	1.87	0.555	0.082
Urinary N excretion, g/d	12.31	9.55	9.72	11.98	10.46	8.55	8.73	1.46	0.138	0.814
Fecal N excretion, g/d	7.37	11.14	11.00	11.03	14.92	12.41	11.37	1.19	0.005	0.013
Whole-body N retention, g/d	26.40	27.68	26.65	23.86	24.78	26.90	27.67	1.87	0.970	0.250

^1^Maximum value for the standard error of the means.

^2^ *P*-values for linear or quadratic contrast effects of the treatments.

^3^Sows with low feed consumption or with incomplete urine collection were excluded from the analysis.

**Table 5.** Nitrogen utilization in gestating gilts fed diets containing 0.10%, 0.13%, 0.17%, 0.20%, 0.23%, 0.27,%, or 0.30% SID Met between days 109 and 112 of gestation

	SID Met, %							SEM^1^	*P*-value^2^	
	0.10	0.13	0.17	0.20	0.23	0.27	0.30		Linear	Quadratic
Period 4 (days 109 to 112)										
Number of sows^3^	8	9	10	9	10	10	9			
N intake, g/d	71.51	70.30	73.97	70.53	72.39	72.49	74.56			
Total N excretion, g/d	42.49	29.29	31.31	29.36	29.12	28.18	34.61	2.42	0.006	< 0.001
Urinary N excretion, g/d	19.47	10.38	11.89	11.61	13.11	13.21	17.68	2.02	0.874	< 0.001
Fecal N excretion, g/d	22.86	18.74	20.20	17.50	15.83	15.72	16.09	0.79	< 0.001	0.011
Whole-body N retention, g/d	29.02	41.02	42.66	41.17	43.28	44.31	39.95	2.42	< 0.001	< 0.001

^1^Maximum value for the standard error of the means.

^2^ *P*-values for linear or quadratic contrast effects of the treatments.

^3^Sows with low feed consumption or with incomplete urine collection were excluded from the analysis.

### Plasma methionine metabolites

**Figure 2.**
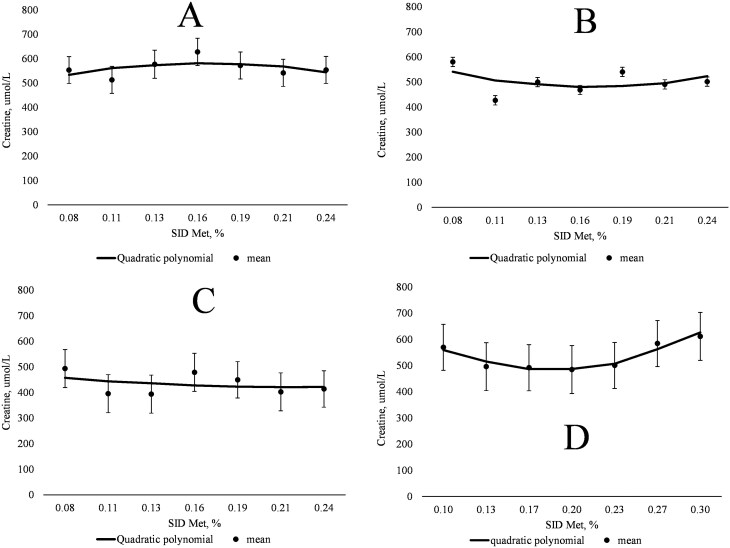
(A) Quadratic polynomial model for the relationship between plasma creatine and SID Met (% of diet) of gilts on day 38 of gestation (*n* = 10 for each treatment). Plasma creatine, µmol/L = 401.3 + 2,181.6 × (SID Met, %) + (−6,606.8) × (SID Met, %)^2^; Bayesian information criterion (BIC) = 902.9; plasma creatine optimized at 0.17% SID Met. (B) Quadratic polynomial model for the relationship between plasma creatine and SID Met (% of diet) of gilts on day 53 of gestation (left to right; *n* = 10, 9, 9, 10, 10, 9, and 10 respectively). Plasma creatine, µmol/L = 705.6 + (−2,704.3 × SID Met, %) + 8,098.9 × (SID Met, %)^2^; BIC = 904.3; inflection point at 0.17% SID Met. (C) Quadratic polynomial model for the relationship between plasma creatine and SID Met (% of diet) of gilts on day 87 of gestation (left to right; *n* = 10, 9, 9, 9, 10, 10, and 10 respectively). Plasma creatine, µmol/L = 510.5 + (−811.4 × SID Met, %) + 1,838.7 × (SID Met, %)^2^; BIC = 885.1; Inflection point at 0.22% SID Met. (D) Quadratic polynomial model for the relationship between plasma creatine and SID methionine (% of diet) of gilts on day 109 of gestation (left to right; *n* = 8, 9, 10, 9, 10, 10, and 9 respectively). Plasma creatine, µmol/L = 842.2 + (−3,887.3) × (SID Met, %) + 10,559 × (SID Met, %)^2^; BIC = 932; Inflection point at 0.18% SID Met.

**Figure 3.**
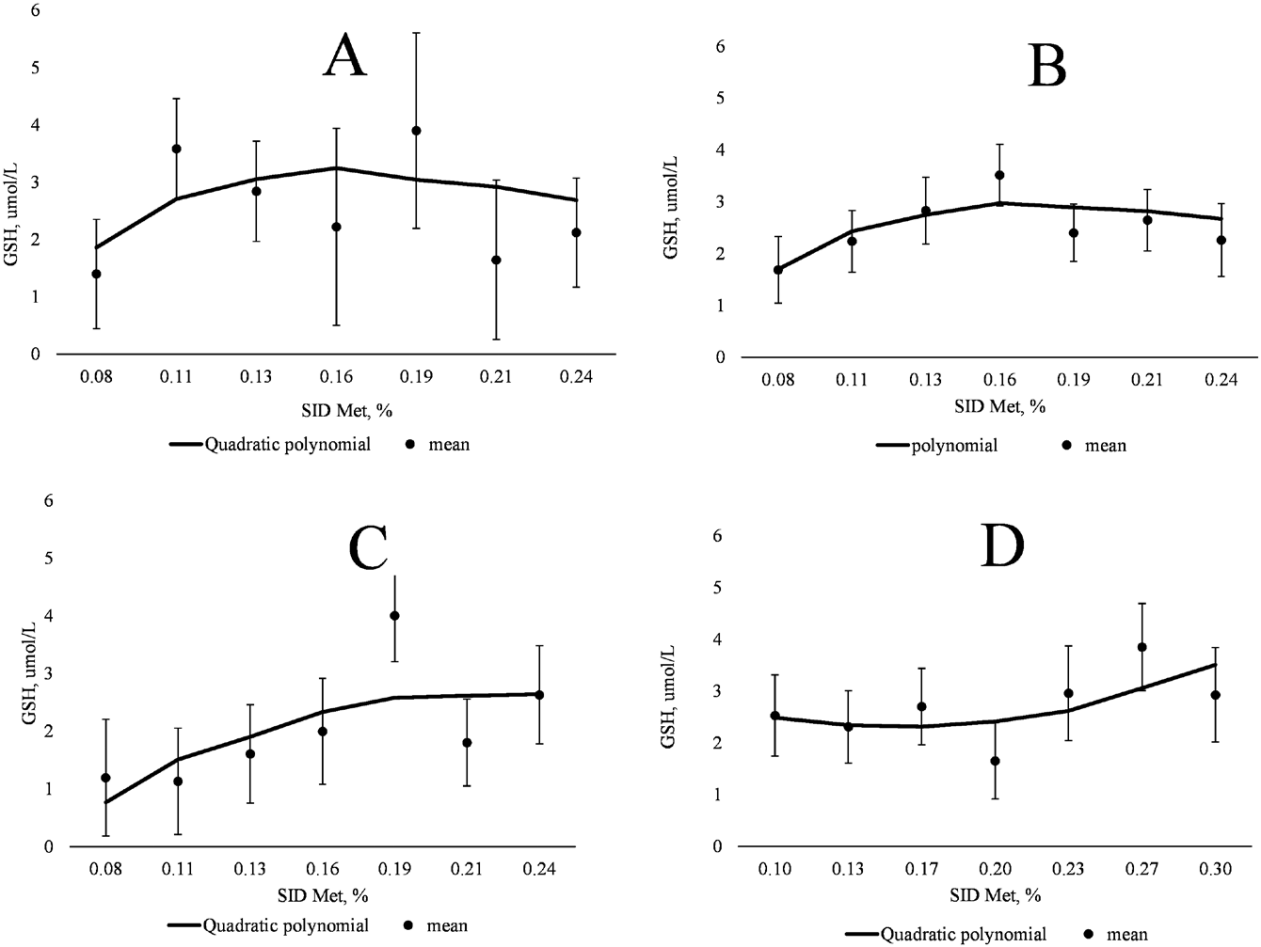
(A) Quadratic polynomial model for the relationship between plasma glutathione (GSH) and SID Met (% of diet) of gilts on day 38 of gestation (*n* = 10 for each treatment). Plasma GSH, µmol/L = −2.325 + 69.8 × (SID Met, %) + (−218.8) × (SID Met, %)^2^; Bayesian information criterion (BIC) = 155; plasma GSH optimized at 0.16% SID Met. (B) Quadratic polynomial model for the relationship between plasma GSH and SID Met (% of diet) of gilts on day 53 of gestation (left to right; *n* = 10, 9, 9, 10, 10, 9, and 10 respectively). Plasma GSH, µmol/L = −1.7 + (56.5 × SID Met, %) + (−169.2) × (SID Met, %)^2^; BIC = 184.6; plasma GSH optimized at 0.17% SID Met. (C) Quadratic polynomial model for the relationship between plasma GSH and SID Met (% of diet) of gilts on day 87 of gestation (left to right; *n* = 10, 9, 9, 9, 10, 10, and 10 respectively). Plasma GSH, µmol/L = −2.1 + (44.5 × SID Met, %) + (−103.8) × (SID Met, %)^2;^ BIC = 221.6; Inflection point at 0.21% SID Met. (D) Quadratic polynomial model for the relationship between plasma GSH and SID methionine (% of diet) of gilts on day 109 of gestation (left to right; *n* = 8, 9, 10, 9, 10, 10, and 9 respectively). Plasma GSH, µmol/L = 3.7 + (−18.2) × (SID Met, %) + 58.4 × (SID Met, %)^2^; BIC = 210.4; Inflection point at 0.16% SID Met.

On days 38, 53, and 87, the plasma concentrations of Hcys, cysteinyl-glycine, creatine, Tau, and SUN did not exhibit a linear or quadratic relationship with increasing SID Met content (Table 6). The QPM indicated inflection points for plasma creatine at 0.17%, 0.17%, and 0.22% SID Met (3.8, 3.8, and 4.9 g SID Met/d) on days 38, 53, and 87, respectively (Fig. 2, panels A, B, and C, respectively). Plasma concentrations of GSH tended to increase then decrease (quadratic; *P *= 0.096) as dietary SID Met increased on day 53, whereas GSH tended to increase (linear; *P *= 0.095) as dietary SID Met increased on day 87. The QPM indicated inflection points for plasma GSH at 0.16%, 0.17%, and 0.21% SID Met (3.5, 3.8 and 4.6 g SID Met/d) on days 38, 53, and 87, respectively (Fig. 3, panels A, B, and C, respectively).

**Table 6.** Plasma metabolite concentrations and serum urea nitrogen in gestating gilts fed experimental diets containing 0.08%, 0.11%, 0.13%, 0.16%, 0.19%, 0.21%, or 0.24% SID Met

	SID Met, %							SEM^1^	*P*-value^2^	
	0.08	0.11	0.13	0.16	0.19	0.21	0.24		Linear	Quad
*Metabolite, µmol/L* ^3^										
Day 38										
Number of sows^4^	10	10	10	10	10	10	10			
Homocysteine	17.86	21.69	18.9	16.96	17.31	17.54	18.13	1.67	0.215	0.781
Cysteinyl-glycine	17.07	15.5	15.38	16.36	17.14	16.78	17.26	1.34	0.369	0.361
Glutathione	1.42	3.62	2.82	2.23	3.87	1.62	2.08	1.69	0.867	0.307
Creatine	553	513	577	628	572	542	554	55	0.825	0.355
Taurine	89.80	92.10	103.30	85.50	121.50	96.01	105.20	14.83	0.166	0.642
Urea N (mmol/L)	4.02	4.48	5.94	4.42	4.28	4.26	4.74	0.53	0.964	0.266
Met	35	37	37	40	39	33	40	3	0.591	0.711
Cys	26	33	18	32	27	20	34	6	0.844	0.460
Day 53										
Number of sows^4^	10	9	9	10	10	9	10			
Homocysteine	16.99	16.96	15.47	17.61	17.17	15.04	16.34	1.32	0.523	0.879
Cysteinyl-glycine	16.26	16.064	14.99	15.16	16.72	14.81	16.63	1.56	0.967	0.504
Glutathione	1.57	2.17	2.70	3.52	2.36	2.59	2.29	0.80	0.432	0.096
Creatine	580	427	500	468	540	490	501	80	0.834	0.411
Taurine	94.84	101.01	107.20	104.56	83.30	90.70	98.33	14.10	0.533	0.779
Urea N (mmol/L)	3.99	4.43	3.97	4.66	5.17	3.71	4.88	0.43	0.215	0.696
Met	34	34	35	35	32	39	35	3	0.491	0.819
Cys	37	33	29	27	45	29	37	5	0.785	0.320
Day 87										
Number of sows^4^	10	9	9	9	10	10	10			
Homocysteine	21.17	19.14	20.61	21.05	23.85	23.59	23.82	4.49	0.184	0.781
Cysteinyl-glycine	19.84	19.74	17.85	19.07	22.41	22.02	23.08	4.41	0.216	0.506
Glutathione	1.09	1.22	1.57	1.88	3.95	1.81	2.57	1.03	0.095	0.485
Creatine	494	395	394	479	449	402	413	74	0.610	0.830
Taurine	91.97	95.14	86.39	97.58	97.7	89.6	82.4	9.29	0.470	0.303
Urea N (mmol/L)	4.03	3.27	4.17	4.36	4.09	4.16	4.05	0.36	0.339	0.644
Met	32	37	33	35	35	34	36	3	0.777	0.918
Cys	28	44	36	37	29	33	27	5	0.219	0.150

^1^Maximum value for the standard error of the means.

^2^ *P*-values for linear or quadratic contrast effects of the treatments.

^3^Blood samples were collected after a 7-d adaptation period, 15 h after the last meal.

^4^Sows with low feed consumption or with incomplete urine collection were excluded from the analysis.

**Figure 4.**
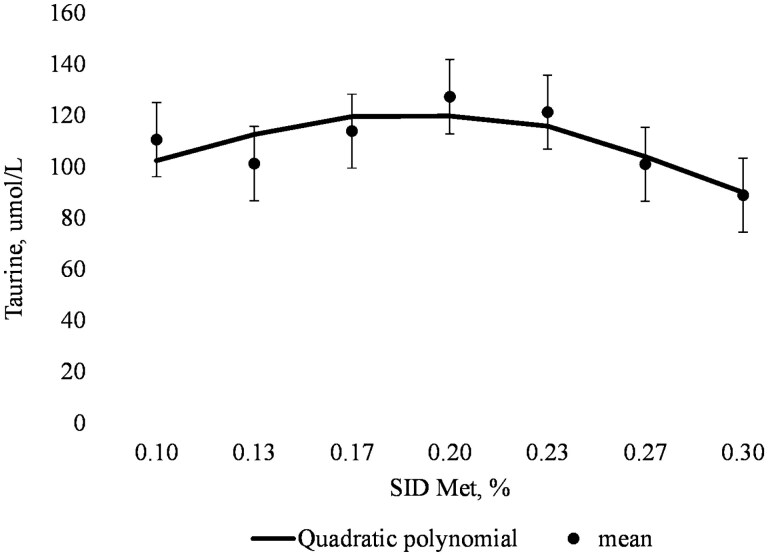
Quadratic polynomial model for the relationship between plasma taurine (Tau) and SID Met (% of diet) of gilts on day 109 of gestation (left to right; *n* = 8, 9, 10, 9, 10, 10, and 9 respectively). Plasma Tau, µmol/L = 37.6 + 883.8 × (SID Met, %) + (−2,364.3) × (SID Met, %)^2^; Bayesian information criterion (BIC) = 641.7; plasma Tau optimized at 0.19% SID Met.

On day 109, plasma concentrations of Hcys, cysteinyl-glycine, creatine, GSH, and SUN did not exhibit a linear or quadratic relationship with increasing SID Met content, but plasma concentrations of Tau increased then decreased (quadratic; *P *< 0.05) as dietary SID Met increased (Table 7). The QPM indicated inflection points for plasma creatine, GSH, and Tau at 0.18% (4.7 g SID Met/d), 0.16% (4.2 g SID Met/d), and 0.19% (5.0 g SID Met/d), respectively (Fig. 2 panel D, Fig. 3 panel D, and Fig. 4, respectively).

**Table 7.** Plasma metabolite concentrations and serum urea nitrogen in gestating gilts fed experimental diets containing 0.10%, 0.13%, 0.17%, 0.20%, 0.23%, 0.27%, or 0.30% SID Met

	SID Met, %							SEM^1^	*P*-value^2^	
	0.10	0.13	0.17	0.20	0.23	0.27	0.30		linear	quad
*Metabolite, µmol/L* ^3^										
Day 109										
Number of sows^4^	8	9	10	9	10	10	9			
Homocysteine	16.41	19.39	18.61	18.53	19.27	17.67	18.38	0.99	0.520	0.105
Cysteinyl-glycine	15.29	16.99	17.87	17.76	16.26	16.36	18.81	0.95	0.130	0.739
Glutathione	2.52	2.33	2.66	1.69	2.76	3.81	3.05	0.99	0.255	0.503
Creatine	570	496	492	484	501	584	611	92	0.412	0.138
Taurine	110	101	114	127	121	101	89	18	0.269	0.036
Urea N (mmol/L)	5.56	4.10	4.61	4.46	5.06	4.90	4.93	0.47	0.944	0.120
Met	29	31	30	32	32	29	28	3	0.523	0.232
Cys	32	25	21	25	39	29	29	8	0.733	0.740

^1^Maximum value for the standard error of the means.

^2^ *P*-values for linear or quadratic contrast effects of the treatments.

^3^Blood samples were collected after a 7-d adaptation period, 15 h after the last meal.

^4^Sows with low feed consumption or with incomplete urine collection were excluded from the analysis.

### Plasma AA profile

On day 38, fasted plasma concentrations of Ile decreased then increased (quadratic; *P *< 0.05), total indispensable AA increased (linear; *P *< 0.05), and Phe, Gln, Ser, and total dispensable AA tended to increase (linear; *P *= 0.076, *P *= 0.068, *P *= 0.058, and *P *= 0.050, respectively) with increasing dietary SID Met content; the remaining AA were not influenced by dietary treatment (Table S1). On day 53, fasted plasma concentrations of Phe increased then decreased (quadratic; *P *< 0.05) and Tyr increased (linear and quadratic; *P *< 0.05) with increasing dietary SID Met content; the remaining AA were not influenced by dietary treatment (Table S2). On day 87, plasma concentrations of Thr increased (linear; *P *< 0.05) with increasing dietary SID Met content, while the remaining AA were not influenced by dietary treatment (Table S3). On day 109, His increased then decreased (quadratic; *P <* 0.05), Ile tended to increase then decrease (quadratic; *P *= 0.081), Gln increased (linear; *P *< 0.001), and total dispensable AA tended to increase (linear; *P *= 0.050) with increasing dietary SID Met content; the remaining AA were not influenced by dietary treatment (Table S4).

## Discussion

The objective of the present study was to evaluate the current NRC (2012) recommendation for Met for gilts throughout gestation using whole-body N retention and to describe the impact of dietary Met inclusion level on indicators of Met utilization for roles beyond protein synthesis. Though the addition of dl-Met was not balanced with Ala or another N source, the diets were considered isonitrogenous and isocaloric since the N and energy provided by dl-Met was marginal (i.e., up to 0.39 g/d for periods 1, 2, and 3, and 0.56 g/d for period 4 of additional N, which was less than 1% of total daily N supply). Despite some deviation in the analyzed vs. expected Met contents, total dietary Met supply increased with enough separation among treatments to allow interpretation of the response variables in a dose–response study.

For whole-body N retention, the break-point analysis indicated minimum SID Met requirements of 3.8, 4.2, 3.5, and 6.0 g SID Met/d between gestation days 38 and 41, 53 and 56, 87 and 90, and 109 to 112. Therefore, in all periods of gestation tested in the current study, the calculated minimum SID Met requirements were above the NRC (2012) SID Met recommendations of 3.5 g SID Met/d before day 90 of gestation and 5.2 g SID Met/d after day 90 after gestation. Thus, it appears that current genetics with greater prolificacy have 8% greater SID Met needs for N retention in early-to-mid pregnancy and 13% greater SID Met needs in late pregnancy than estimated by the NRC (2012) gestating sow model.

The use of break-point models allows for determination of either the maximum response of the variable (optimum response) or the inflection point after which the variable starts to improve in response increasing SID Met supply. Several studies have reported an improvement in N retention when AA are titrated in the diets of swine, as increasing supply of the first limiting AA will increase protein retention and reduce oxidation and N excretion from excess unbalanced AA (Kerr and Easter, 1995; Toledo et al., 2014; Spring et al., 2020). It is pertinent to highlight the N retention results in periods 1 and 3 occurred as inflection points while responses more similar to those observed in periods 2 and 4 were expected in the current study. Others have demonstrated that whole-body N retention is dynamic and that the prioritization between retention in maternal and pregnancy-associated pools shifts throughout gestation (e.g., Dourmad et al., 1996; NRC, 2012; Miller et al., 2016). Since the pregnancy-associated pools are relatively well characterized and exponential fetal growth is a major contributor to whole-body N retention in late gestation, the estimate of Met requirements between day 109 and 112 of gestation using N balance techniques is considered the most reliable. Conversely, AA utilization for protein deposition is more poorly characterized in early and mid-gestation during placenta formation and expansion (starting at d18 of gestation; Wu et al., 2017), generation of additional pregnancy-associated structures like the allantois and yolk sac (after day 18 of gestation; Bertasoli et al., 2015), implantation (between days 30 and 60; Almeida and Dias, 2022), synthesis and secretion of amniotic and allantoic fluids (between days 30 and 60; Vallet et al., 2009), and implementation of nutrient transport systems to the fetus (between days 80 and 105; Almeida and Dias, 2022). In addition, the utilization of Met for protein synthesis and other nonprotein fates are intertwined, especially during instances of rapid growth. For example, Met is needed to generate SAM, which can be used for polyamine synthesis, while polyamines are necessary for cell growth but have also been shown to support increased protein synthesis (Wu et al., 2013). Moreover, the neonatal piglet has significant need to expand nonprotein pools including creatine and PC, which use a significant proportion of Met supply (McBrearity et al., 2013; Robinson et al., 2016a); the same is likely true for fetal piglets, especially in late gestation, though such partitioning has not been quantified previously. Therefore, it is possible that the balance between protein synthesis and Met utilization for nonprotein fates could have influenced the whole-body N retention observed in the current study. Finally, the lack of differences in plasma Hcys demonstrated that the effect of dietary Met in the synthesis of nonprotein molecules may be expressed in other parts of the pathways as Met metabolism is cyclical and Hcys is a central molecule that can be either a precursor or a product, depending on the portion of the cycle that is most active.

In the current study, circulating concentrations of plasma creatine did not respond strongly to maternal dietary Met supply within gestation stage. During gestation in pigs, it is not known if creatine is synthesized within the fetus or if creatine is supplied by maternal circulation, placing the metabolic burden of creatine synthesis on the sow. It has been shown however, that creatine supply during gestation is particularly relevant for ATP production in the creatine kinase circuit and as a preventative mechanism against hypoxia-ischemia in the fetus (Berger et al., 2004; Muccini et al., 2021). Fetal supply of creatine in other species widely varies with some demonstrating increased maternal creatine synthesis and placental transport during pregnancy and others demonstrating that the fetus is competent in creatine synthesis by mid-gestation (e.g., Koszalka et al., 1972; Ellery et al., 2015; Sah et al., 2023). The locations of creatine synthesis and utilization may have impacted the ability to detect changes in (maternal) plasma creatine concentrations in response to dietary Met supply. It has been proposed previously that the circulating plasma concentrations of creatine may be used as indicator of methyl status, as creatine synthesis consumes a significant proportion of the methyl groups from SAM in transmethylation reactions (Mudd et al., 2007). Moreover, creatine synthesis occurs in liver, necessitating transport via the circulatory system to other tissues (Brosnan et al., 2011). Based on the break-point analyses, the maximum plasma creatine concentration was achieved at 3.8 g SID Met/d on day 38 of gestation. Conversely, for days 53 and 109 of gestation, the inflection points indicate ascending concentrations of plasma creatine occurred after 3.8 and 4.0 g SID Met/d, respectively, which suggested that the SID Met inclusion to maximize maternal plasma creatine concentration may be beyond the highest SID Met inclusion in the current experiment. Although an inflection point was detected on day 87 as well, it was nearly beyond the highest SID Met inclusion. Therefore, the maternal supply of dietary Met required to maximize creatine concentrations in maternal circulation is at least similar to the Met supply needed to maximize whole-body N retention, though the characterization of creatine accumulation in tissues, creatine kinase activity, and Met flux toward transmethylation reactions during pregnancy are needed.

In the current study, the response of maternal plasma GSH concentration to increasing dietary supply of SID Met was variable among gestation periods with optimal dietary Met indicated at 3.5, 3.8, and 4.6 g SID Met/d on days 38, 53, and 87 of gestation, respectively. An inflection point was observed at 4.2 g SID Met/d on day 109 of gestation, indicating that the dietary inclusion of SID Met to optimize maternal plasma GSH concentration in late gestation may be beyond the highest inclusion used in the current experiment. A high (plasma) concentration of GSH is often associated with a greater capacity to combat oxidative agents at the cellular level (Njälsson and Norgren, 2005). An important factor in GSH synthesis is the sufficient availability of Cys that can originate directly from the diet or protein breakdown or be synthesized endogenously from Hcys via the transsulfuration pathway. Since exogenous Cys was included in the diet with the intention of minimizing the need of dietary Met for transsulfuration, it is possible that plasma GSH concentration was not strongly sensitive to SID Met supply. Moreover, plasma concentrations of AA that constitute GSH (i.e., Cys, Gly, and Glu) were not influenced by SID Met supply, possibly since blood collection was conducted 15 h after the previous meal. Stable isotope tracer studies and assessment of enzyme activities related to the synthesis of GSH could provide further insight into Met kinetics for transsulfuration and GSH synthesis when the supply of the precursor (Met) is altered. Conversely, and only in late gestation (day 109), plasma Tau demonstrated a very strong response to dietary SID Met supply, whereby plasma Tau concentration was maximized at an apparent lower dietary SID Met content than whole-body N retention (5.0 vs. 6.0 g SID Met/d, respectively). It is possible that Tau may play an important role during gestation due to its high concentration in placental fluid and its uptake by the fetuses at the end of gestation, as described in other species (Aerts and Van Assche, 2002). Moreover, Tau is necessary for the development and maintenance of neurological functions (Della Corte et al., 2002) and, in weaning piglets, improves intestinal barrier function (Yang and Liao, 2019). The relevance of alterations in plasma Tau concentrations for the pregnant gilt and the developing fetuses before and after birth have yet to be determined.

It is possible that another mathematical model may better fit the response variables in the current study. In the paper by Ramirez-Camba and Levesque (2023), the authors reported 2 inflection points for dietary AA depending on perceived physiological need of the animal: 1 for protein retention and 1 that was described as the optimal AA supply for other key metabolic functions related to survival (e.g., relating to milk yield, litter size, immune response etc.), the latter of which occurred at a higher dietary AA inclusion and at the expense of N utilization efficiency. Since Met has roles of quantitative significance beyond protein synthesis, such a model may also be appropriate for the current dataset. Combined, the results of the current study and the recently published arguments by Ramirez-Camba and Levesque (2023) encourage a reassessment of how nutritionists define AA requirements for livestock production species.

Finally, the unexpected response of fecal N excretion to SID Met supply in each period suggests that the inclusion of SID Met may influence N endogenous losses and thus, influence the calculation of whole-body N retention. There are a number of factors that have been reported to alter endogenous N losses (Adeola et al., 2016; Acosta et al., 2017; Soomro et al., 2017), but Met has not been yet identified as a contributing factor for either specific or basal endogenous losses. In addition, analytical recovery of the indigestible marker in the diets could have contributed to the observed differences in fecal N output. Despite an average marker recovery of 99% among the diets, the CV was 7% for the diets fed in periods 1, 2, and 3, and 12% for the diets fed in period 4. Moreover, the lowest marker recovery for the diets fed in periods 1 to 3 occurred for the lowest SID Met diet (0.08% SID Met) and the highest marker recovery for the diets fed in period 4 also occurred for the lowest SID Met diet (0.10% SID Met), which could have contributed to the linear responses observed for fecal N excretion relating to dietary Met content and a slight overestimation (periods 1 to 3) and underestimation (period 4) of whole-body N retention.

## Conclusion

The current SID Met feeding recommendations provided in the NRC (2012) gestating sow model are likely insufficient to maximize whole-body N retention of gilts at all stages of gestation in current production conditions. The supply of SID Met might also influence Met utilization for metabolic fates beyond protein retention in the gestating gilt. Therefore, future work should quantify the utilization of Met for protein synthesis vs. flux toward the transmethylation pathway and subsequent effects on lactation performance, future reproduction and longevity of the sow, and offspring growth performance, to confirm whether these nonprotein uses of Met should be considered in Met feeding recommendations for gestating gilts.

## Supplementary Material

skaf156_suppl_Supplementary_Materials

## Abbreviations

AA: amino acid
ATTD: apparent total tract digestibility
BIC: bayesian information criterion
BLQ: broken-line quadratic
GSH: glutathione
Hcys: homocysteine
PC: phosphatidylcholine
QPM: quadratic polynomial model
SAA: sulfur-containing AA
SAM: s-adenosyl-methionine
SID: standardized ileal digestible
SUN: serum urea nitrogen
Tau: taurine
UPLC: ultra-performance liquid chromatography
