# Supplementary material for: Estimating standardized ileal digestible methionine requirements for gilts during gestation using whole-body nitrogen retention and describing plasma creatine, glutathione, and taurine concentrations
Source: J Anim Sci. 2025 May 4;103:skaf156. doi: 10.1093/jas/skaf156 (PMC12147025; doi:10.1093/jas/skaf156)
Supplement: skaf156_suppl_Supplementary_Materials [file skaf156_suppl_supplementary_materials.docx]

**Supplementary table 1**. Plasma amino acid concentrations in gestating gilts on d 38 fed experimental diets containing 0.08%, 0.11%, 0.13%, 0.16%, 0.19%, 0.21%, or 0.24 % standardized ileal digestible Met

|  | Standardized ileal digestible Met, % | | | | | | | SEM^1^ | *P*- value^2^ | |
| --- | --- | --- | --- | --- | --- | --- | --- | --- | --- | --- |
|  | 0.08 | 0.11 | 0.13 | 0.16 | 0.19 | 0.21 | 0.24 |  | linear | quad |
| Indispensable AA, uM^3^ | | |  |  |  |  |  |  |  |  |
| Arg | 242 | 231 | 247 | 224 | 224 | 234 | 256 | 14 | 0.723 | 0.118 |
| His | 104 | 101 | 98 | 108 | 141 | 107 | 117 | 15 | 0.158 | 0.709 |
| Ile | 126 | 125 | 128 | 111 | 128 | 132 | 143 | 10 | 0.083 | 0.041 |
| Leu | 200 | 238 | 208 | 201 | 225 | 214 | 224 | 14 | 0.532 | 0.855 |
| Lys | 241 | 263 | 235 | 231 | 242 | 253 | 262 | 17 | 0.538 | 0.257 |
| Met | 35 | 37 | 37 | 40 | 39 | 33 | 40 | 3 | 0.591 | 0.711 |
| Phe | 84 | 88 | 82 | 87 | 108 | 92 | 92 | 7 | 0.076 | 0.472 |
| Thr | 205 | 227 | 219 | 239 | 241 | 229 | 241 | 30 | 0.199 | 0.563 |
| Trp | 65 | 60 | 65 | 57 | 60 | 59 | 67 | 5 | 0.965 | 0.207 |
| Val | 375 | 404 | 376 | 368 | 380 | 397 | 408 | 18 | 0.224 | 0.182 |
| Total | 1,707 | 1,757 | 1,687 | 1,690 | 1,739 | 1,764 | 1,894 | 92 | 0.045 | 0.076 |
| Dispensable AA, uM | | |  |  |  |  |  |  |  |  |
| Ala | 384 | 397 | 464 | 418 | 387 | 387 | 375 | 35 | 0.371 | 0.081 |
| Asn | 59 | 67 | 59 | 70 | 83 | 71 | 78 | 20 | 0.173 | 0.884 |
| Asp | 15 | 20 | 17 | 15 | 18 | 18 | 16 | 2 | 0.853 | 0.389 |
| Cys | 26 | 33 | 18 | 32 | 27 | 20 | 34 | 6 | 0.844 | 0.460 |
| Gln | 1,289 | 1,455 | 1,441 | 1,452 | 1,478 | 1,377 | 1,527 | 73 | 0.068 | 0.395 |
| Glu | 175 | 209 | 185 | 185 | 184 | 210 | 191 | 15 | 0.416 | 0.896 |
| Gly | 1,192 | 1,133 | 1,375 | 1,255 | 1,119 | 1,247 | 1,329 | 86 | 0.377 | 0.889 |
| Pro | 216 | 228 | 234 | 218 | 294 | 227 | 230 | 27 | 0.480 | 0.356 |
| Ser | 137 | 158 | 152 | 161 | 180 | 187 | 180 | 21 | 0.058 | 0.782 |
| Tyr | 82 | 80 | 76 | 74 | 106 | 83 | 85 | 9 | 0.338 | 0.895 |
| Total | 3,523 | 3,766 | 4,027 | 4,052 | 3,915 | 3,795 | 4,030 | 142 | 0.050 | 0.079 |

^1^ Maximum value for the standard error of the means.

^2^ *P*-values for linear or quadratic contrast effects of the treatments.

^3^ Blood samples were collected after a seven-day adaptation period, 15 h after the last meal.

**Supplementary table 2**. Plasma amino acid concentrations in gestating gilts on d 53 fed experimental diets containing 0.08%, 0.11%, 0.13%, 0.16%, 0.19%, 0.21%, or 0.24 % standardized ileal digestible Met

|  | Standardized ileal digestible Met, % | | | | | | | SEM^1^ | *P*- value^2^ | |
| --- | --- | --- | --- | --- | --- | --- | --- | --- | --- | --- |
|  | 0.08 | 0.11 | 0.13 | 0.16 | 0.19 | 0.21 | 0.24 |  | linear | quadratic |
| Indispensable AA, uM^3^ | |  |  |  |  |  |  |  |  |  |
| Arg | 248 | 224 | 221 | 258 | 236 | 240 | 229 | 17 | 0.899 | 0.904 |
| His | 93 | 92 | 94 | 105 | 91 | 95 | 99 | 8 | 0.539 | 0.797 |
| Ile | 143 | 133 | 137 | 132 | 141 | 134 | 128 | 8 | 0.165 | 0.852 |
| Leu | 210 | 205 | 211 | 207 | 203 | 205 | 200 | 12 | 0.321 | 0.737 |
| Lys | 258 | 243 | 254 | 219 | 242 | 252 | 229 | 21 | 0.274 | 0.591 |
| Met | 34 | 34 | 35 | 35 | 32 | 39 | 35 | 3 | 0.491 | 0.819 |
| Phe | 79 | 82 | 84 | 85 | 81 | 78 | 76 | 4 | 0.219 | 0.038 |
| Thr | 234 | 245 | 244 | 277 | 235 | 226 | 253 | 51 | 0.931 | 0.550 |
| Trp | 60 | 58 | 59 | 67 | 57 | 61 | 55 | 5 | 0.555 | 0.272 |
| Val | 400 | 379 | 387 | 374 | 375 | 408 | 372 | 23 | 0.701 | 0.644 |
| Total | 1,807 | 1,698 | 1,712 | 1,748 | 1,704 | 1,744 | 1,666 | 107 | 0.279 | 0.815 |
| Dispensable AA, uM | |  |  |  |  |  |  |  |  |  |
| Ala | 459 | 378 | 385 | 424 | 421 | 414 | 385 | 43 | 0.383 | 0.641 |
| Asn | 92 | 117 | 88 | 96 | 78 | 92 | 66 | 25 | 0.113 | 0.540 |
| Asp | 40 | 76 | 64 | 67 | 65 | 37 | 81 | 45 | 0.535 | 0.694 |
| Cys | 37 | 33 | 29 | 27 | 45 | 29 | 37 | 5 | 0.785 | 0.320 |
| Gln | 1,338 | 1,356 | 1,354 | 1,423 | 1,380 | 1,352 | 1,285 | 63 | 0.674 | 0.171 |
| Glu | 224 | 221 | 226 | 199 | 198 | 197 | 207 | 26 | 0.100 | 0.517 |
| Gly | 1,316 | 1,268 | 1,296 | 1,397 | 1,259 | 1,322 | 1,255 | 57 | 0.687 | 0.411 |
| Pro | 231 | 210 | 203 | 231 | 221 | 223 | 224 | 12 | 0.605 | 0.290 |
| Ser | 169 | 154 | 163 | 175 | 159 | 158 | 150 | 9 | 0.149 | 0.288 |
| Tyr | 80 | 81 | 81 | 89 | 82 | 74 | 70 | 4 | 0.031 | 0.008 |
| Total | 3,972 | 3,598 | 3,787 | 3,818 | 3,906 | 3,897 | 3,687 | 158 | 0.813 | 0.953 |

^1^ Maximum value for the standard error of the means.

^2^ *P*-values for linear or quadratic contrast effects of the treatments.

^3^ Blood samples were collected after a seven-day adaptation period, 15 h after the last meal.

**Supplementary table 3**. Plasma amino acid concentrations in gestating gilts on d 87 fed experimental diets containing 0.08%, 0.11%, 0.13%, 0.16%, 0.19%, 0.21%, or 0.24 % standardized ileal digestible Met

|  | Standardized ileal digestible Met, % | | | | | | | SEM^1^ | *P*- value^2^ | |
| --- | --- | --- | --- | --- | --- | --- | --- | --- | --- | --- |
|  | 0.08 | 0.11 | 0.13 | 0.16 | 0.19 | 0.21 | 0.24 |  | linear | quadratic |
| Indispensable AA, uM^3^ | |  |  |  |  |  |  |  |  |  |
| Arg | 256 | 245 | 232 | 236 | 246 | 244 | 240 | 18 | 0.625 | 0.452 |
| His | 101 | 102 | 93 | 94 | 95 | 102 | 99 | 6 | 0.901 | 0.175 |
| Ile | 141 | 125 | 132 | 147 | 117 | 132 | 130 | 11 | 0.556 | 0.844 |
| Leu | 204 | 172 | 182 | 185 | 190 | 194 | 186 | 12 | 0.931 | 0.213 |
| Lys | 270 | 171 | 218 | 234 | 234 | 220 | 227 | 18 | 0.857 | 0.174 |
| Met | 32 | 37 | 33 | 35 | 35 | 34 | 36 | 3 | 0.777 | 0.918 |
| Phe | 90 | 85 | 81 | 83 | 80 | 86 | 80 | 5 | 0.218 | 0.419 |
| Thr | 199 | 163 | 173 | 181 | 219 | 228 | 189 | 19 | 0.049 | 0.759 |
| Trp | 66 | 57 | 54 | 65 | 59 | 62 | 60 | 4 | 0.961 | 0.380 |
| Val | 377 | 357 | 353 | 374 | 362 | 364 | 358 | 21 | 0.661 | 0.799 |
| Total | 1,657 | 1,487 | 1,501 | 1,540 | 1,565 | 1,632 | 1,558 | 81 | 0.849 | 0.180 |
| Dispensable AA, uM | |  |  |  |  |  |  |  |  |  |
| Ala | 453 | 492 | 454 | 444 | 495 | 508 | 466 | 63 | 0.571 | 0.934 |
| Asn | 83 | 94 | 109 | 88 | 100 | 103 | 96 | 17 | 0.504 | 0.469 |
| Asp | 15 | 16 | 14 | 13 | 14 | 16 | 14 | 1 | 0.722 | 0.236 |
| Cys | 28 | 44 | 36 | 37 | 29 | 33 | 27 | 5 | 0.219 | 0.150 |
| Gln | 1,244 | 1,118 | 1,361 | 1,127 | 1,216 | 1,163 | 1,211 | 71 | 0.684 | 0.958 |
| Glu | 221 | 201 | 189 | 185 | 184 | 211 | 193 | 19 | 0.407 | 0.144 |
| Gly | 1,312 | 1,479 | 1,355 | 1,354 | 1,366 | 1,432 | 1,439 | 95 | 0.475 | 0.804 |
| Pro | 250 | 223 | 215 | 204 | 244 | 237 | 225 | 17 | 0.772 | 0.100 |
| Ser | 164 | 166 | 151 | 149 | 156 | 165 | 163 | 12 | 0.999 | 0.116 |
| Tyr | 86 | 81 | 84 | 77 | 85 | 88 | 85 | 5 | 0.657 | 0.359 |
| Total | 3,804 | 3,776 | 4,014 | 3,700 | 3,871 | 4,002 | 3,915 | 189 | 0.456 | 0.924 |

^1^ Maximum value for the standard error of the means.

^2^ *P*-values for linear or quadratic contrast effects of the treatments.

^3^ Blood samples were collected after a seven-day adaptation period, 15 h after the last meal.

**Supplementary table 4**. Plasma amino acid concentrations in gestating gilts on d 109 fed experimental diets containing 0.10%, 0.13%, 0.17%, 0.20%, 0.23%, 0.27%, or 0.30 % standardized ileal digestible Met

|  | Standardized ileal digestible Met, % | | | | | | | SEM^1^ | *P*- value^2^ | |
| --- | --- | --- | --- | --- | --- | --- | --- | --- | --- | --- |
|  | 0.10% | 0.13% | 0.17% | 0.20% | 0.23% | 0.27% | 0.30% |  | linear | quadratic |
| Indispensable AA, uM^3^ | |  |  |  |  |  |  |  |  |  |
| Arg | 219 | 217 | 225 | 227 | 202 | 233 | 212 | 16 | 0.829 | 0.784 |
| His | 108 | 98 | 110 | 141 | 130 | 103 | 93 | 21 | 0.825 | 0.018 |
| Ile | 159 | 149 | 158 | 172 | 169 | 151 | 151 | 13 | 0.846 | 0.081 |
| Leu | 175 | 174 | 174 | 192 | 169 | 189 | 184 | 15 | 0.267 | 0.990 |
| Lys | 180 | 196 | 177 | 235 | 180 | 203 | 208 | 19 | 0.324 | 0.692 |
| Met | 29 | 31 | 30 | 32 | 32 | 29 | 28 | 3 | 0.523 | 0.232 |
| Phe | 88 | 83 | 88 | 93 | 96 | 90 | 86 | 8 | 0.617 | 0.266 |
| Thr | 168 | 173 | 231 | 173 | 153 | 181 | 190 | 18 | 0.978 | 0.738 |
| Trp | 59 | 54 | 59 | 54 | 56 | 61 | 59 | 4 | 0.495 | 0.340 |
| Val | 407 | 405 | 417 | 423 | 403 | 410 | 418 | 29 | 0.686 | 0.859 |
| Total | 1,577 | 1,558 | 1,624 | 1,627 | 1,500 | 1,597 | 1,569 | 89 | 0.767 | 0.718 |
| Dispensable AA, uM | |  |  |  |  |  |  |  |  |  |
| Ala | 364 | 536 | 442 | 450 | 486 | 458 | 472 | 60 | 0.311 | 0.263 |
| Asn | 119 | 91 | 114 | 91 | 117 | 97 | 92 | 24 | 0.381 | 0.975 |
| Asp | 98 | 67 | 69 | 86 | 160 | 79 | 71 | 67 | 0.801 | 0.392 |
| Cys | 32 | 25 | 21 | 25 | 39 | 29 | 29 | 8 | 0.733 | 0.740 |
| Gln | 729 | 1,119 | 833 | 903 | 1,060 | 1,134 | 1,144 | 68 | < 0.001 | 0.905 |
| Glu | 201 | 210 | 171 | 224 | 210 | 191 | 201 | 25 | 0.987 | 0.880 |
| Gly | 1,191 | 1,392 | 1,422 | 1,304 | 1,181 | 1,200 | 1,317 | 118 | 0.603 | 0.572 |
| Pro | 222 | 235 | 233 | 241 | 242 | 233 | 251 | 21 | 0.300 | 0.902 |
| Ser | 192 | 173 | 198 | 218 | 199 | 179 | 188 | 21 | 0.970 | 0.338 |
| Tyr | 123 | 83 | 88 | 107 | 88 | 79 | 83 | 18 | 0.176 | 0.665 |
| Total | 3,156 | 3,543 | 3,414 | 3,267 | 3,315 | 3,644 | 3,799 | 212 | 0.050 | 0.393 |

^1^ Maximum value for the standard error of the means.

^2^ *P*-values for linear or quadratic contrast effects of the treatments.

^3^ Blood samples were collected after a seven-day adaptation period, 15 h after the last meal.

**Complementary equations:**

*Gestation d 38 to 41*

Broken-line linear predictive equation for N retention (g/d):

(1) N retention, g/d = 21.7187 – (–39.8846) × (0.08 – SID met, %) if SID met, % < 0.08

21.7187 g/d if SID met, % > 0.08

Bayesian information criterion (BIC)= 416.10

Broken-line quadratic predictive equation for N retention (g/d):

(2) N retention, g/d = 21.7837 + ( –34.6955) × (0.08 – SID met, %) + (500.05) × (0.08 – SID met, %)^2^ if SID met, % < 0.08

21.7837 if SID met, % > 0.08

Bayesian information criterion (BIC)= 417.50

Broken-line linear predictive equation for creatine, µmol/L:

(3) Plasma creatine, µmol/L = 572.93 + (– 457.79) × (0.16 – SID met, %) if SID met, % < 0.16

572.93 if SID met, % > 0.16

Bayesian information criterion (BIC)= 903.2

Broken-line quadratic predictive equation for creatine, µmol/L:

(4) Plasma creatine, µmol/L = 574.47 + (– 5019.8) × (0.1289 – SID met, %) + (93885) × (0.1289 – SID met, %)^2^ if SID met, % < 0.1289

574.47 if SID met, % > 0.1289

Bayesian information criterion (BIC)= 904.5

Broken-line linear predictive equation for GSH, µmol/L:

(5) Plasma GSH, µmol/L = 2.6294 + (– 17.027) × (0.1099 – SID met, %) if SID met, % < 0.1099

2.6294 if SID met, % > 0.1099

Bayesian information criterion (BIC)= 155.8

Broken-line quadratic predictive equation for GSH, µmol/L:

(6) Plasma GSH, µmol/L = 2.5868 + (– 3.5777) × (0.1275 – SID met, %) + (–10.018) × (0.1275 – SID met, %)^2^ if SID met, % < 0.1275

2.5868 if SID met, % > 0.1275

Bayesian information criterion (BIC)= 157.2

*Gestation d 53 to 56*

Quadratic polynomial predictive equation for N retention (g/d):

(7) N retention, g/d = 21.7297 + 8.016 × (SID met, %) – 11.7369 × (SID met, %)^2^

Bayesian information criterion (BIC)= 356.16

Inflexion point at – 0.34 % SID Met.

Broken-line linear predictive equation for N retention (g/d):

(8) N retention, g = 23.507 – (–200.9) × (0.08513 – SID met, %) if SID met, % < 0.08513

23.507 if SID met, % > 0.08513

Bayesian information criterion (BIC)= 357

Broken-line linear predictive equation for creatine, µmol/L:

(9) Plasma creatine, µmol/L = 488.87 + 33267 × (0.08275 – SID met, %) if SID met, % < 0.08275

488.87 if SID met, % > 0.08275

Bayesian information criterion (BIC)= 906.70

Broken-line quadratic predictive equation for creatine, µmol/L:

(10) Plasma creatine, µmol/L = 489.22 + 35886 × (0.08254 – SID met, %) + 13174 × (0.08254 – SID met, %)^2^ if SID met, % < 0.08254

489.22 if SID met, % > 0.08254

Bayesian information criterion (BIC)= 905.40

Broken-line linear predictive equation for GSH, µmol/L:

(11) Plasma GSH, µmol/L = 2.7444 + (– 21.2405) × (0.13 – SID met, %) if SID met, % < 0.13

2.7444 if SID met, % > 0.13

Bayesian information criterion (BIC)= 185.3

Broken-line quadratic predictive equation for GSH, µmol/L:

(12) Plasma GSH, µmol/L = 2.7552 + (– 29.6068) × (0.1299 – SID met, %) + (178.29) × (0.1299 – SID met, %)^2^ if SID met, % < 0.1299

2.7552 if SID met, % > 0.1279

Bayesian information criterion (BIC)= 187.6

*Gestation d 87 to 90*

Broken-line linear predictive equation N retention (g/d):

(13) N retention, g/d = 25.8954 + 15.9526 × (0.16 – SID met, %) if SID met, % < 0.16

25.8954 if SID met, % > 0.16

Bayesian information criterion (BIC)= 378.30

Broken-line quadratic predictive equation N retention (g/d):

(14) N retention, g/d = 26.2456 + (–39.6626) × (0.08 - SID met, %) + 360.02 × (0.08 – SID met, %)^2^ if SID met, % < 0.0.08

26.2456 if SID met, % > 0.08

Bayesian information criterion (BIC)= 381.00

Broken-line linear predictive equation for creatine, µmol/L:

(15) Plasma creatine, µmol/L = 428.19 + (–550253) × (0.08 – SID met, %) if SID met, % < 0.08

428.19 if SID met, % > 0.08

Bayesian information criterion (BIC)= 915.20

Broken-line quadratic predictive equation for creatine, µmol/L:

(16) Plasma creatine, µmol/L = 432.20 + (– 424.55) × (0.08 – SID met, %) + 5902.10 × (0.08 – SID met, %)^2^ if SID met, % < 0.08

432.20 if SID met, % > 0.08

Bayesian information criterion (BIC)= 887.70

Broken-line linear predictive equation for GSH, µmol/L:

(17) Plasma GSH, µmol/L = 2.3989 + (– 29.935) × (0.149 – SID met, %) if SID met, % < 0.149

2.3989 if SID met, % > 0.149

Bayesian information criterion (BIC)= 224.8

Broken-line quadratic predictive equation for GSH, µmol/L:

(18) Plasma GSH, µmol/L = 2.186 + 0.3037 × (0.24 – SID met, %) + (–10.323) × (0.24 – SID met, %)^2^ if SID met, % < 0.24

2.186 if SID met, % > 0.24

Bayesian information criterion (BIC)= 229

*Gestation d 109 to 112*

Broken-line linear predictive equation N retention (g/d):

(19) N retention, g/d = 42.3272 + (–399.17) × (0.1331 – SID met, %) if SID met, % < 0.1331

42.3272 if SID met, % > 0.1331

Bayesian information criterion (BIC)= 379.50

Broken-line quadratic predictive equation N retention (g/d):

(20) N retention, g/d = 42.3272 + (–391.05) × (0.1331 – SID met, %) + (–223.92) × (0.1331 – SID met, %)^2^ if SID met, % < 0.1331

42.3272 if SID met, % > 0.1331

Bayesian information criterion (BIC)= 381.8

Broken-line linear predictive equation for creatine, µmol/L:

(21) Plasma creatine, µmol/L = 527.82 + 5250.32 × (0.108 – SID met, %) if SID met, % < 0.108

527.82 if SID met, % > 0.108

Bayesian information criterion (BIC)= 935.50

Broken-line quadratic predictive equation for creatine, µmol/L:

(22) Plasma creatine, µmol/L = 527.72 + 51001 × (0.1008 – SID met, %) + 14369 × (0.1008 – SID met, %)^2^ if SID met, % < 0.1008

527.72 if SID met, % > 0.1008.

Bayesian information criterion (BIC)= 938

Broken-line linear predictive equation for GSH, µmol/L:

(23) Plasma GSH, µmol/L = 2.6212 + (– 26.9681) × (0.1048 – SID met, %) if SID met, % < 0.1048

2.6212 if SID met, % > 0.1048

Bayesian information criterion (BIC)= 212.50

Broken-line quadratic predictive equation for GSH, µmol/L:

(24) Plasma GSH, µmol/L = 3.4058 + (– 23.4877) × (0.27 – SID met, %) + 111.26 × (0.24 – SID met, %)^2^ if SID met, % < 0.27

3.4058 if SID met, % > 0.27

Bayesian information criterion (BIC)= 211.70

Broken-line linear predictive equation for Tau, µmol/L:

(25) Plasma Tau, µmol/L = 109.05 + 1887.94 × (0.101 – SID met, %) if SID met, % < 0.101

109.05 if SID met, % > 0.101

Bayesian information criterion (BIC)= 647.90
